# Dhat Syndrome: Unusual Manifestation in a Distinct Cultural Context

**DOI:** 10.7759/cureus.73821

**Published:** 2024-11-16

**Authors:** Rihab Farooq, Hanana Arif, Ammu Thulaseedharan, Ammara Shakil Sahibole

**Affiliations:** 1 Psychiatry, Al Amal Psychiatric Hospital, Emirates Health Services, Dubai, ARE; 2 General Practice, Abeer AlNoor Polyclinic, Dubai , ARE; 3 Psychiatry and Behavioral Sciences, Al Amal Psychiatric Hospital, Emirates Health Services, Dubai, ARE; 4 General Practice, GSM Medical Center, Dubai, ARE

**Keywords:** anxiety, cultural psychiatry, dhat, dhat syndrome, psychiatry

## Abstract

Dhat syndrome is a culture-bound syndrome that presents with concerns about semen loss. This is a case of a 38-year-old Indian male who was brought to our psychiatric emergency department in Dubai, United Arab Emirates due to concerns of dizziness, excessive worry, and preoccupation with health. He reported somatic symptoms of left lumbar pain and passage of small, white stones during urination a few weeks before presentation. Subsequently, he developed multiple other symptoms like palpitations, tremors, sweating, and a few fainting episodes among others. During the assessment, it was understood he believed he was losing semen and this was affecting him significantly. He also had a few other stressors in his personal life. He previously had similar symptoms and claimed that traditional treatments treated him, hence insisted on a traditional healer at this time. He was found to have an anxious mood on further examination and was diagnosed with Dhat syndrome. He was started on treatment with a benzodiazepine and an antidepressant. This case piqued our interest as it is a condition with a significant cultural basis which presented in a region outside the relevant cultural region. The symptoms the patient presented with and the concerns he was seeking treatment for were quite unusual and seemed a rare incidence in the Middle East region.

## Introduction

We report a typical case of Dhat, a “culture-bound syndrome,” yet atypical in terms of geographic and cultural prevalence. Dhat finds its origins in Hindu Ayurvedic medicine which considers semen to be one of the vital fluids, to the extent that ancient Vedic literature depicts it as “the force of life” [1]. As per the Diagnostic and Statistical Manual of Mental Disorders, Fifth Edition (DSM-5), it falls under cultural formulation interview, yet due to its strong comorbidities, some consider it a variant of depression with a 40-66% prevalence, while others look at it as a variant of somatization disorder with a prevalence of 40% [2,3]. Anxiety disorders were also found in 21-38% of patients [2,3]. Accounting for 76.7% of all culture-bound syndromes, Dhat is found to be the most common [4]. 

While most patients prefer to seek help from indigenous practitioners, psychiatrically the treatment modalities are based on the comorbid symptoms of depression and anxiety, in addition to psychological counseling techniques [3].

Due to globalization and cultural exchange, encountering patients with culturally bound syndromes in any place of the world is no longer a rarity, as is the case of our patient.

Similar to other somatization disorders, Dhat syndrome may be erroneously perceived as a urological or medical condition, leading to a substantial investment of time and resources in diagnostic investigations. The primary objective is to enhance knowledge and awareness of Dhat syndrome among psychiatrists, with the ultimate goal of reducing the time from presentation to diagnosis.

## Case presentation

We report the case of a 38-year-old male originating from the Indian subcontinent who presented to our psychiatric emergency in Dubai, United Arab Emirates. The patient was residing in Dubai and had been working as a fabricator for the last four years.

He was brought in by his company’s medical in-charge with complaints of dizziness, worry, and excessive preoccupation with his health. On further questioning, he revealed that he experienced symptoms of left colicky lumbar region pain, along with the passage of stones during urination two to three weeks before presentation. As per the patient's description, the stones were approximately 1-2 mm in size, white in color, and smooth in consistency. Following this, he began to experience episodes of palpitations, breathlessness, excessive sweating, hand tremors, numbness in his hands and feet, classic sensations of impending doom, headache, and pressure-like sensations. As confirmed by the medical in-charge who accompanied him, he also had two to three fainting episodes. These episodes would generally last 5-10 minutes before settling on their own. Over the last few days, they had increased in frequency and had begun to affect the patient’s functionality causing him to stop reporting to work. The company's medical staff reported that he repeatedly continued to visit the staff clinic with these complaints despite reassurance that his blood and urine investigations were normal. Mid-interview, the patient got nervous and demanded a male doctor. When informed of the availability constraints, he explained to us that he believes the stones he has passed are semen that has solidified. In addition, he also believes that he is losing semen through his urine. He felt that this loss of semen was draining his body of energy and blood.

The patient reported having recently gotten married in his country back home and was noticed to be constantly seeking reassurance that his fertility would not be affected. He was also undergoing financial stress and had large sums of loans to pay back which he had borrowed for the wedding. His mother was also recently diagnosed with cancer, and he had been worried about not being able to afford treatment.

Furthermore, he revealed experiencing similar complaints two years ago, where he had passed stones. He then visited a urologist and was prescribed allopathic treatment. He also visited a traditional healer and was prescribed pills and essential oils. About two to three weeks after initiating treatment, his symptoms subsided, and he attributed the cure to the traditional healers’ medications. Currently, as well, the patient was noted to be seeking reassurance and insisting that we do not have his cure at our facility and that he needs to see a traditional healer.

Mental state examination was significant for anxious mood and affect, no perceptual abnormalities or thought process and content impairments were identified. We proceeded to diagnose the patient as Dhat syndrome due to his superstitious beliefs that correlated semen loss with loss of energy and vitality. Consequently, he was started on clonazepam 0.5 mg BID along with sertraline 25 mg OD.

After this encounter the patient was unfortunately lost to follow-up.

## Discussion

The story of Dhat dates many centuries back originating in the Indian subcontinent when it was believed that our bodies consisted of seven different body fluids [5] and out of these, semen was the most valuable one. Concepts originating in Ayurvedic literature talked about the formation of semen by a process of purification and condensation through several steps. “During each step of this process there occurs forty times condensation and ultimately one drop of semen is formed from 40 drops of bone marrow”. In addition, it was mentioned that “semen attributes to physical beauty, physical strength, and mental strength” [6].

Ultimately these concepts led to the belief that loss of excessive semen in any form whether through pre-marital intercourse, masturbation, nocturnal emissions, or loss through urine or stool is harmful [7,8]. This resulted in stress which amplified, and symptoms took the shape of “semen loss anxiety,” depression, somatoform disorders, psychosexual disorders, and hypochondriasis [3].

Unfortunately, traditional healers, quacks, and alternative medicine practitioners took advantage of this belief and the resulting symptoms and thus started to advertise and propagate their treatment modalities. This began to be advertised everywhere on walls, on television, in newspapers, and on roadside hoardings in most northern Indian cities. To date, studies have shown that most patients with psychosexual disorders reach general medical practitioners, practitioners of alternative medicine, and traditional healers, instead of approaching a psychiatrist for consultation [9].

It is the widespread publicity of these beliefs that then led to further mitigation, distortion, and dissemination of this message and the further arousal of myths and distorted beliefs. It was not until the 1960s that Dr. Wig, a renowned psychiatrist in India introduced the term "Dhat syndrome" [10], and the term was formally used for the first time in scientific literature.

In the international classificatory systems, for the first time, Dhat syndrome got a place as a diagnosis entity, in the International Classification of Diseases and Health Related Conditions, 10th Edition (ICD-10) [11] and the Fourth edition of the Diagnostic and Statistical Manual of Mental Disorders (DSM-IV) [12]. As for the DSM-5 (APA, 2013) it includes "dhat syndrome" in its Glossary of Cultural Concepts of Distress. 

Treatment modalities mainly focus on initiating the diagnostic process by excluding medical co-morbidities and sexual disorders. In view of the symptoms of anxiety, depression, and deranged thinking processes the mainstay of treatment involves the use of antidepressants, anxiolytics, and in some countries, even antipsychotics the use of which is much debated [13]. Cognitive behavior therapy (CBT) and psychotherapeutic modalities have also been employed. One study in the literature concluded that CBT was indeed effective and perhaps equivalent to medication or placebo in the treatment of Dhat [14]. Yet the study had its limitations in terms of having a very small sample size, subjects chosen were not fully representative of the classic model of presentation of Dhat syndrome.

It was expected that with the inclusion in the ICD-10 and DSM-IV, the debate on its diagnosis would end, however, it continued for years and is still ongoing. Many people still question its liability as a distinct diagnosis and many others question its overlap with depression, anxiety, and somatic symptom disorder [12]. Many still argue that it can be equivalent to depression and may be used as a specifier of depression, or it may be a cultural way of manifesting distress [2]. Some have even gone to the extent of questioning it being a delusion in itself [15].

## Conclusions

In the context of our patient, what stood out was the distinctive manner in which he presented his symptoms. He linked the passing of kidney stones to the loss of semen, a correlation that has not been documented in current literature. Typically, most patients report the spontaneous loss of semen, either through urine or during activities such as masturbation or intercourse, among others, as previously discussed.

Another aspect we seek to emphasize in this case is that in an era of extensive globalization, cultural syndromes are no longer confined to specific cultures. To our knowledge, this is the first reported case of Dhat syndrome identified in the United Arab Emirates. This underscores the importance of psychiatrists having comprehensive knowledge and awareness of all cultural manifestations of illness to diagnose and treat patients effectively. Without this understanding, such patients might have been misdiagnosed or misunderstood by various medical professionals across the field, including general practitioners, urologists, specialists in sexually transmitted diseases, and others.
